# General anesthesia with cisatracurium and sevoflurane for a patient with primary carnitine deficiency receiving L-carnitine therapy: A case report

**DOI:** 10.1097/MD.0000000000027333

**Published:** 2021-09-24

**Authors:** Ling Ai, Yan Fang

**Affiliations:** Department of Anesthesiology, Tongji Hospital, Tongji Medical College, Huazhong University of Science and Technology, Wuhan, People's Republic of China.

**Keywords:** cisatracurium, L-carnitine, lipid storage myopathies, primary carnitine deficiency, sevoflurane

## Abstract

**Rationale:**

Lipid storage myopathies (LSMs) are a series of genetic disorders of lipid metabolism predominantly affecting muscle. The low incidence and lethal properties of this disease make anesthesia experience limited in such patients. Among all etiologies of LSMs, primary carnitine deficiency (PCD) is now considered highly treatable by early administration of L-carnitine, though it remains unclear whether L-carnitine is effective enough to protect diseased muscle against conventionally used neuromuscular blocking agents (NMBAs) during general anesthesia. Currently, no data are available concerning possible prolonged muscle weakness in these cases.

**Patient concerns:**

This case presents a 43-year-old female who was diagnosed with a PCD-induced LSM 3 years ago due to fatigability and exertional myalgias and has been treated with L-carnitine ever since. At the time of this report, she was admitted for uterine fibroids and scheduled for selective open gynecologic surgery under general anesthesia.

**Diagnosis:**

The patient's diagnosis of PCD-induced LSM was based on the clinical features, muscle biopsy, and diminished organic cation/carnitine transporter 2 (OCTN2) transporter activity in the patient's skin fibroblasts.

**Interventions:**

L-carnitine was taken by the patient until the morning of surgery. General anesthesia with cisatracurium and sevoflurane was selected as the anesthetic plan during the operation. The train-of-four (TOF) test was adopted as additional monitoring, particularly to track the recovery of neuromuscular function.

**Outcomes:**

The patient was extubated successfully following a spontaneously restored TOF ratio (TOFR) of 0.9. Nonetheless, we recorded a prolonged efficacy of cisatracurium in the clinical duration and the recovery time with TOFRs of 0.7 and 0.9, respectively.

**Lessons:**

The conventional dose of cisatracurium combined with a low dose of sevoflurane can be safely used in patients with LSMs without additional anesthetic risks. Meanwhile, continuous TOF monitoring is recommended to perform high-quality anesthesia.

## 1 Introduction

Lipid storage myopathies (LSMs) are defined as a group of genetic disorders characterized by impaired lipid β-oxidation and excessive triglyceride droplet accumulation in muscle. As one of the four etiological factors of LSMs, primary carnitine deficiency (PCD) is caused by dysfunctional organic cation/carnitine transporter 2 (OCTN2) due to a mutation in the SLC22A5 gene.^[1]^ The subsequent inability of lipids to fuel as alternative sources of energy can be associated with many metabolic disorders of fatty acids. Lipid metabolic organs such as the heart, liver, brain and skeletal muscle, are major targets of this pathological alteration, where skeletal muscle is most commonly involved. Patients suffering from PCD usually present with myopathic manifestations such as fluctuating weakness, hypotonia, myalgia, and abnormal fatigability.^[2–4]^ When anesthesia is required for such patients, the pre-existing myopathy and underlying multiple organ insufficiencies must be taken into account, and considerably more attention should be given to those issues during anesthesia management.

However, reports on the anesthesia experience with PCD patients are very limited due to its low incidence (1:40,000 to 1:120,000).^[5,6]^ Although progressive PCD leads to life-threatening complications such as respiratory muscle weakness, L-carnitine supplementation is extremely effective in correcting and maintaining organ function within normal limits for a long time.^[7–9]^ Owing to a longer life expectancy with the treatment of L-carnitine, patients with PCD may experience more surgical possibilities. Furthermore, well-tolerated general anesthesia would enhance their chances of being treated by surgery successfully, and this is urgently needed in this population. However, as conventional general anesthetics, neuromuscular blocking agents (NMBAs) have long been questioned for their safety in patients with myopathy, mainly due to the risk of prolonged postoperative muscle weakness, which may result from the susceptibility of damaged muscle tissues to NMBAs. In this case, cisatracurium was chosen among multiple NMBAs, for its unique Hofmann elimination, which implicates spontaneous degradation in plasma and tissues. We hypothesized that the long-term regular administration of L-carnitine would be effective in combating pre-existing myopathy in this patient, thus ensuring a normal metabolic rate of cisatracurium. To validate the hypothesis, train-of-four (TOF) and other related NMBA pharmacodynamic indices were recorded as the criteria for extubation. In addition, we chose sevoflurane for intraoperative anesthesia maintenance to reduce the dose of NMBA.

In the present study, we described the case of anesthesia management in a PCD patient under the context of L-carnitine treatment and highlighted the efficiency and safety of cisatracurium combined with a low dose of sevoflurane on pre-existing myopathy in this patient.

## 2 Methods

Ethical review and approval were not required for this study on a human participant in accordance with the local legislation and institutional requirements. The patient provided written informed consent to participate in this study.

## 3 Case report

**Figure 1:**
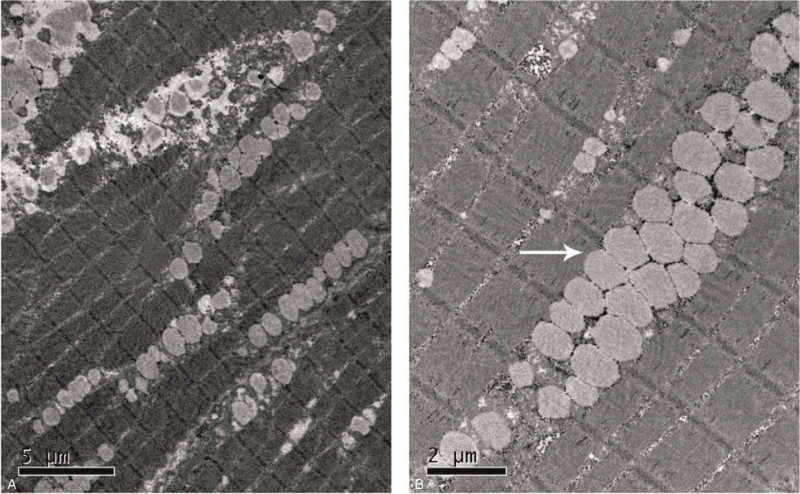
Electron microscopy images of the patient's skeletal muscle biopsy. (A) shows the extensive distribution of triglycerides in the skeletal muscle fibers; the arrow in (B) indicates the abnormally accumulated lipid droplets in the muscle.

A 43-year-old female patient was scheduled for selective panhysterectomy owing to uterine fibroids. Her symptoms of fatigability and exertional myalgias began 3 years ago. A histochemical examination displayed pronounced features of a LSM characterized by the appearance of abnormal storage of triglyceride droplets in muscle fibers (Fig. 1). No other family members or immediate relatives showed similar symptoms except for her younger sister. The diagnosis of PCD was subsequently established according to the diminished OCTN2 transporter activity in the patient's skin fibroblasts. The genetic analysis could not be conducted due to her sister's unwillingness to participate. The patient reported a dramatic improvement in muscle strength after taking L-carnitine (1 g bid, orally). A physical examination revealed the patient's muscle strength was at level 3/5 in her limbs, while no apparent atrophy was observed. There were no obvious abnormalities shown by preoperative chest radiography, electrocardiogram or echocardiogram. In addition, the routine laboratory findings including the results of liver and renal function tests were all within normal limits.

**Figure 2:**
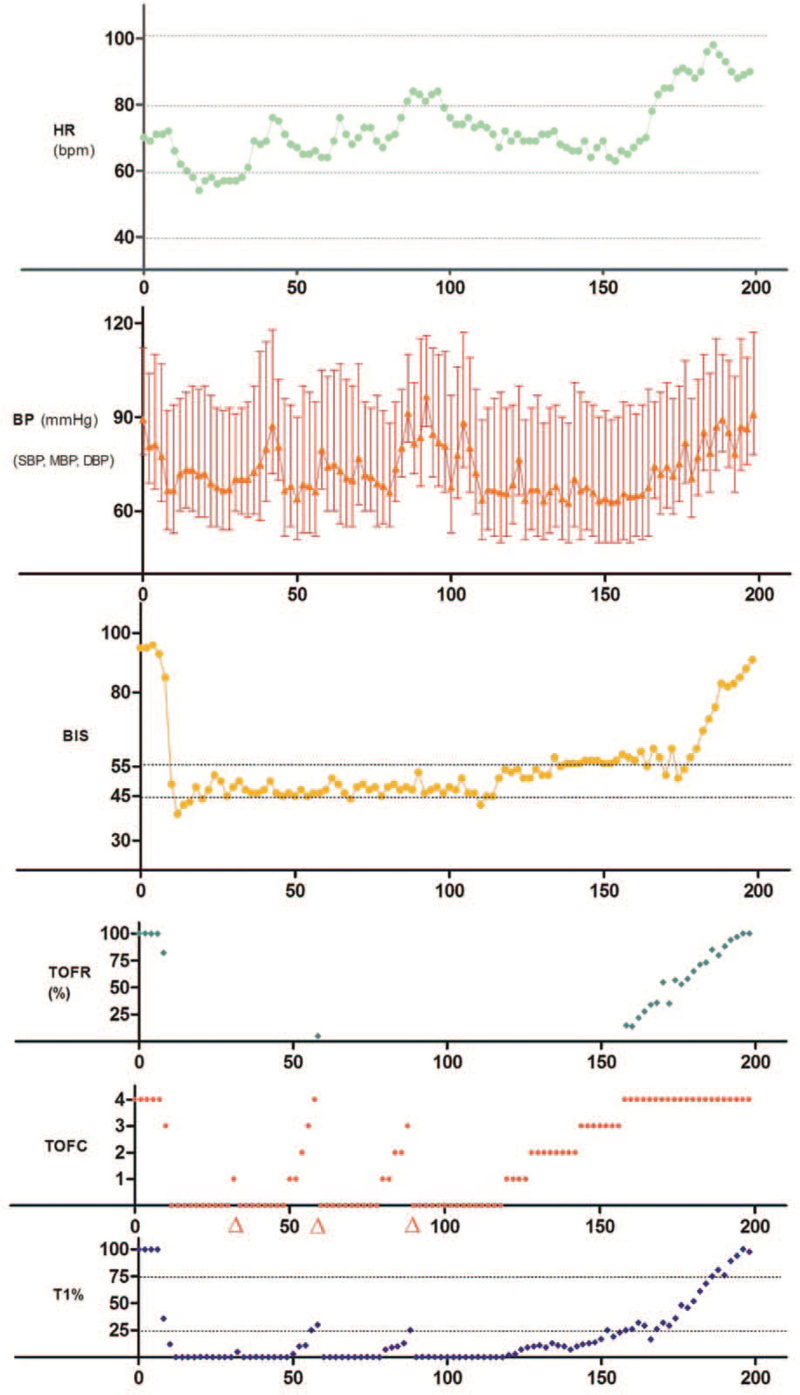
The heart rate (HR), blood pressure (BP), and depth of anesthesia were recorded throughout the entire general anesthesia, as well as the changes in neuromuscular function monitored by train-of-four (TOF). 251658240^Δ^ represents the three repeated doses of cisatracurium; BIS = bispectral index, bpm = beats per minute, T_1_ = the percent of T_1_ returned to the baseline, TOFC = train-of-four count, TOFR = train-of-four ratio.

L-carnitine was administered as usual on the morning of the surgery. Upon the patient's arrival in the operating room, routine intravenous access and noninvasive monitoring were established. To monitor the intraoperative arterial pressure and serum glucose, a 22-gauge radial arterial cannula was inserted by puncturing the left forearm before anesthetic induction. The patient's preoperative blood glucose was 5.2 mmol/L. Intraoperative monitoring included non-invasive blood pressure (NIBP), heart rate (HR), pulse oximetry (SpO2), electrocardiography (ECG), end-tidal carbon dioxide levels, and core temperature. Additionally, the bispectral index (BIS) was adopted for anesthesia depth monitoring and TOF was used for neuromuscular function assessment. After the patient was preoxygenated, general anesthesia was induced with sufentanil (6 μg/kg) followed by propofol (2 mg/kg). Continuous monitoring with TOF (Mindray 3-directional neuromuscular transmission transducer acceleromyography module, Shenzhen, China) was performed through supramaximal ulnar nerve stimulation (2 Hz/2 s per 2 min) as described by Naguib et al.^[10]^ After achieving a stable baseline measurement, an initial dose of cisatracurium (0.15 mg/kg) was injected to facilitate tracheal intubation. The protocol for anesthesia maintenance consisted of remifentanil (0.15 μg/kg/min) and sevoflurane (0.88∼1.71 minimum alveolar concentration, MAC) mixed with 60% air oxygen, while the BIS was maintained between 45–55. The patient received a total of three repeated doses of cisatracurium (0.05 mg/kg). One dose was given when the first twitching response of the TOF (T1) attained only 5% control due to the surgeon's complaint of abdominal tension; another two doses were administered when T1 attained 25% control. All three doses of cisatracurium led to 100% neuromuscular blockade. After the last injection, four pharmacodynamic parameters of cisatracurium were recorded. The results showed that the clinical duration (return of T1 to 25% baseline), recovery index (T1 25%–75%), time to achieve T4/T1 ratio (TOFR) of 0.7 and 0.9 of cisatracurium were 77 min, 21 min, 94 min and 101 min, respectively (Table 1). Figure 2 summarizes the entire process of neuromuscular function change occurred during surgery. The entire surgical procedure lasted 162 min, during which the patient's intraoperative serum glucose was consistently above 5.0 mmol/L and her core temperature remained stable at 36.4°C. The patient was successfully extubated following spontaneously restored TOFR to 0.9 even in the absence of the antagonist neostigmine. Thereafter, the patient was transferred to the postoperative anesthesia care unit for further evaluation. No perioperative or anesthetic complications occurred. Finally, the patient was discharged home on the fifth postoperative day.

**Table 1:** Recovery criteria of cisatracurium when co-administrate with sevoflurane [time data present as mean (SD)].

Author	Patient groups	Clinical duration (T_1_ 25%; min)	Recovery index (T_1_25%-75%; min)	TOFR = 0.7 (min)	TOFR = 0.8 (min)	TOFR = 0.9 (min)	Sevoflurane (MAC)
Our case	Patients with PCD	77	21	94		101	0.88∼1.17
Braga Ade, F. et al 2002^[17]^	Normal population	66.2 (13.42)	23.6 (5.02)				1.17
Cavalcanti, I. L. et al 2002^[18]^	Patients with chronic renal falure	75.4 (24.6)	28.0 (16.7)	51.4 (25.3)			0.29∼0.58
	Control group	68.9 (13.4)	20.2 (12.4)	34.3 (12.4)			0.5∼0.58
Keles, G. T. et al 2004^[16]^	Elderly patients	56 (14)	20 (7.5)		88 (19)		0.58 (with 50% N_2_O/O_2_ mixture)
Soltész, S. et al 2002^[19]^	Infants	55 (13)	21 (14)			73 (15)	1.17
	children	41 (16)	16 (7)			59 (14)	

min = minute, N_2_O = Nitrous Oxide, O_2_ = Oxygen, TOFR = Train-of-four ratio.

## 4 Discussion

PCD is a rare but worldwide autosomal recessive disorder caused by the lack of functional OCTN2 in the human body, which results in a marked decrease in carnitine uptake and an increase in urinary carnitine loss.^[5,6,11,12]^ PCD can be fatal if early diagnosis and intervention do not occur promptly. When the carnitine level is insufficient to assist fatty acids across into the mitochondrial membrane for energy production, organs such as the heart, skeletal muscles, and brain are unable to use fat as spare energy during prolonged aerobic work or fasting. Nevertheless, unusable lipids are still released into plasma during fasting. Their entry into the liver, skeletal muscle, and heart may interfere strongly with the physiological functions of these vital organs. Anesthesiologists should be aware of the potential impact of general anesthesia on PCD patients. To date, there are only a few reported cases concerning the use of NMBAs and inhalation anesthetics in these patient groups. Of the three known case reports, two patients were treated with NMBAs and one of them had received continuous carnitine treatment. Inhalational anesthetic agents were adopted in all cases for anesthesia maintenance. No significant prolonged muscle relaxation times have been reported, and all three anesthetic courses were completely uneventful.^[12–14]^ However, none of the cases reported the exact pharmacodynamics of NMBAs. Our report was the first to provide pharmacodynamic data on cisatracurium in PCD patients in the context of carnitine treatment.

We selected inhaled anesthetics for anesthesia maintenance instead of propofol because the overburdened lipid emulsion infusion may aggravate the risk of lipid intolerance in PCD patients. Additionally, the potential inhibitory effect of propofol on carnitine palmityl transferase I may further exacerbate PCD and PCD-like disorders.^[15]^ In this regard, we suggest that inhaled anesthetics would be the preferred alternative to propofol for anesthesia maintenance. Furthermore, sevoflurane should take priority over other inhaled anesthetics due to its characteristics of rapid induction, recovery, and minimal biotransformation. Moreover, exposure to sevoflurane results in additional potentiation of the efficacy of an NMBA, and patients with LSMs may benefit from a reduced dose of NMBAs. Because the impact of NMBAs on muscle weakness in patients with myopathy is uncertain, nonaccumulative NMBAs, which mainly rely on Hofman elimination, have become the most suitable candidates for PCD patients. When the possible coexisted cardiac complications are taken into consideration, cisatracurium may be the best option for these patients due to the advantages of its modest effect on blood pressure. Our case is the first report of cisatracurium pharmacodynamics under the administration of sevoflurane in patients with PCD. As shown in Table 1, when sevoflurane was constantly administered, the clinical duration, and recovery index of cisatracurium in our case were comparable to the mean values in the normal population, elderly individuals, infants, children and those with chronic renal failure.^[16–19]^ However, at recovery times of TOFRs of 0.7 and 0.9, our data presented a longer recovery profile of cisatracurium. A possible explanation for the discrepancy is that different concentrations of sevoflurane were used across studies. However, to our knowledge, sevoflurane below 2 MAC would not affect the effective dose of cisatracurium with the increased concentration.^[20]^ Given neither the aforementioned studies nor our case used sevoflurane for more than 2 MAC, sevoflurane cannot be the cause of the prolonged recovery time of TOFRs of 0.7 and 0.9 in our case, while, instead, PCD-induced myopathy might be quite relevant for this issue.

As the current gold standard for safe extubation, the spontaneous recovery of neuromuscular function to a TOFR of 0.9, in the absence of the antagonist neostigmine is a preferred method for pharmacodynamics research of NMBAs. In the context of L-carnitine, our patient demonstrated a complete spontaneous recovery from cisatracurium to a TOFR of 0.9. Although this case showed a prolonged recovery time with a TOFR of 0.9, the safety of cisatracurium has been fully demonstrated in such patients.

It is noteworthy that the surgeon complained of transient tensioning of the abdominal muscles when the TOF monitor showed an appropriate value that completely met the requirements for abdominal surgery. PCD-related LSMs may account for this phenomenon. There have been reports of unequal severities of weakness among different body parts in PCD patients, where the neck and limbs were more susceptible to PCD-induced myopathy than the trunk.^[21–23]^ In our case, L-carnitine supplementation was not capable of eliminating this kind of differential myasthenia. Therefore, we recommended multisite neuromuscular function monitoring for these populations, although the ulnar nerve-adductor pollicis unit has long been regarded as the gold standard to evaluate the effects of NMBAs.^[10]^

The various clinical manifestations of PCD range from asymptomatic to sudden infant death. Patients with different ages of onset can have different types of presentation, during which milder clinical types are more frequent in adolescence and adulthood and are mostly associated with a muscular presentation. Deadly cardiac or hepatic symptoms usually occur in childhood.^[24,25]^ Based on the variability in clinical features, this disease can be divided into two groups: systemic carnitine deficiency is characterized by recurrent episodes of hypoglycemia and hepatic and/or cardiac involvement in infancy or early childhood, while myopathic carnitine deficiency is often associated with progressive LSMs in adulthood.^[2]^ Of the patients in the second class, increased serum carnitine concentrations can simultaneously improve skeletal muscle morphology and function. An elevated serum carnitine level after L-carnitine administration is closely associated not only with reduced lipid droplet accumulation in muscles but also with an enhanced functional mitochondrial respiratory chain.^[26–28]^ A 28-year follow-up study showed that regularly taking L-carnitine even allowed a PCD patient for the ability to perform gym-based exercise, which fully demonstrated the ability of L-carnitine to reverse impaired muscle function. Meanwhile, the rapid recurrence of fatigue following the discontinuation of L-carnitine highlights the importance of continuous administration.^[9]^ According to the classification criteria of PCD, our patient had myopathic carnitine deficiency. However, the late-onset or sole presenting myopathic phenotype does not mean that we can simplify the preoperative examination for patients confirmed to have PCD. Electrocardiogram, echocardiography, abdominal ultrasound, and serum transaminase can provide good risk assessments of potentially fatal cardiac and hepatic abnormalities. Unfortunately, our case did not include an extra test for serum creatine kinase to assess the severity of myopathy before surgery. Because advanced lipid deposition myopathy may exhibit a severe muscular atrophy phenotype,^[29]^ we could only infer from the nearly normal function and morphology of the muscle in this case that there was no or only mild muscle impairment and therefore assumed that general anesthesia was safe and feasible, with only a minimal risk of delayed recovery from neuromuscular blocking. This is also a limitation of this case report.

Given that prolonged fasting and surgical stress might have caused our patient to face a glucose shortage and lipolysis initiation, we insisted on regular plasma glucose monitoring during surgery even though no prior symptoms of hypoglycemia had been observed. No episode of intraoperative hypoglycemia occurred in our case despite rapid treatment of glucose-containing intravenous fluids being permitted when necessary. Although oral carnitine supplementation has been reported to successfully maintain appropriate blood concentrations for up to 48 h, we still would like to emphasize the importance of taking the usual daily dose of carnitine until the morning of surgery. Long-term preoperative fasting should be avoided in PCD patients.^[13,30]^

## 5 Conclusions

In summary, this case demonstrated that the long-term administration of L-carnitine provided reassurance on the safety and efficiency of cisatracurium combined with sevoflurane during general anesthesia in a PCD patient. Continuous neuromuscular monitoring was recommended to guide rational NMBA consumption. Additionally, the ulnar nerve-adductor pollocks muscle, as the sole TOF monitoring unit, requires further verification in abdominal surgery, considering that muscle weakness is more severe in the limb muscles than in the abdomens of patients with PCD.

## Acknowledgments

None

## Author contributions

**Conceptualization:** Yan Fang.

**Supervision:** Yan Fang.

**Visualization:** Yan Fang.

**Writing – original draft:** Ling Ai.

**Writing – review & editing:** Yan Fang.
